# Influence of health-related quality of life on health service utilization in Chinese rural-to-urban female migrant workers

**DOI:** 10.1186/s12955-014-0121-4

**Published:** 2014-08-15

**Authors:** Chu-Hong Lu, Pei-Xi Wang, Yi-Xiong Lei, Zhong-Cheng Luo

**Affiliations:** Department of Preventive Medicine, School of Public Health, Guangzhou Medical University, Guangzhou, China; Institute of Public Health, School of Nursing, Henan University, Kaifeng, China; Ministry of Education-Shanghai Key Laboratory of Children’s Environmental Health, Xin Hua Hospital, Shanghai Jiao-Tong University School of Medicine, Shanghai, China

**Keywords:** Health-related quality of life, Health service utilization, Female migrant worker, Chinese

## Abstract

**Background:**

Rural-to-urban migrant workers have been increasing rapidly in China over recent decades. Health related quality of life (HRQOL) may affect health service utilization. There is a lack of data on HRQOL in relation to health service utilization in Chinese rural-to-urban migrant workers. This study was aimed to explore the influence of HRQOL on health service utilization in Chinese rural-to-urban female migrant workers.

**Methods:**

This was a cross-sectional survey of 1,438 female rural-to-urban migrant workers in Shenzhen-Dongguan economic zone, China in 2013. HRQOL was assessed by the 36-items Health Survey Short Form (SF-36). Health service utilization was measured by any physician visit over the recent two weeks and any hospitalization over the last 1-year (annual hospitalization). Clustered logistic regression was used to analyze the influence of HRQOL on health service utilization.

**Results:**

Lower scores in three HRQOL domains (bodily pain, general health, role physical) were associated with more frequent health service utilization in female rural-to-urban migrant workers. Bodily pain and general health were associated with an independent influence of 15.6% on the risk of recent two-week physician visit, while role physical and general health were associated with an independent influence of 21.2% on the risk of annual hospitalization. The independent influence of HRQOL on health service utilization was smaller than that of socio-demographic and health-related variables.

**Conclusions:**

HRQOL may have a modest influence on health service utilization in Chinese rural-to-urban female migrant workers - an underprivileged population in urban China.

## Background

Surging numbers of rural-to-urban migrant workers are a unique phenomenon in China, a result of rapid economic development and urbanization over the last 3 decades. With the rapid development of economy, the migrant population continues to increase from rural to urban areas in China [1,2]. Rural-to-urban migrant population increased from 70 millions in 1993 to 268.9 millions in 2013 [3-5]. Female migrant workers are in high demand as they are generally cheap labor, and particularly suitable for certain jobs requiring dexterity and attention to details (e.g. textile and assembling industries). Female migrant workers play an important role in economic development in China, but they face a variety of social disadvantages in urban areas [6]. The root cause of this phenomenon is the dual household registration system in China. Dual household registration system divides the population into rural and urban citizens. Employment opportunities and social welfare distribution differ according to household registration status. Most social welfare benefits in urban areas are available to registered urban citizens only, but not to registered rural citizens who live in urban areas [1,2,7,8]. The health and quality of life of female migrant workers are an increasingly recognized social concern in China [9]. In general, females tend to have higher health service utilization than males [10-12]. Li H et al. reported that females had higher health service utilization (53.9% vs. 46.2%) than males [11]. Dai M et al. showed that females might have higher recent two-week morbidity (28.8% vs. 24.9%) and higher chronic disease morbidity (35.5% vs. 32.9%) than males [12]. Hence, optimizing the distribution of health service resources to improve the health of migrant female workers has become a priority for public health policy makers [13]. Rural-to-urban migrants in China have been studied in various aspects including socio-demographics, anthropology and management [14-17]. However, to the best of our knowledge (according to literature search in PubMed), there is a lack of data on health related quality of life (HRQOL) in relation to health service utilization in rural-to-urban female migrant workers. HRQOL is an essential aspect of human health embedded in an individual’s physical health, psychological state, social relationships, personal beliefs and relationships to salient features of the environment [18]. Poor HRQOL has been strongly associated with reduced work performance and early retirement. HRQOL is an important issue in caring for the elderly [19,20], acute and chronic disease patients [21,22]. The associations between HRQOL and health service utilization have been reported in chronic disease patients [23,24], aging patients with osteoarthritis [25] and primary care patients [13], and healthy individuals [26]. As an underprivileged population group in China, migrant workers suffer a variety of inequalities including long working hours, insecure employment, overcrowded and insalubrious living conditions [6,27]. Zhu CY et al. founded that HRQOL in rural–urban female migrant workers was lower as compared to Chinese females in the general population [27]. Liu Y et al. showed that HRQOL among migrant workers was significantly lower than governmental civil employees [28]. However, the influence of HRQOL on health service utilization in Chinese female rural-to-urban migrant workers has not yet been reported. We carried out a study to explore the influence of HRQOL on health service utilization in rural-to-urban female migrant workers in Shenzhen-Dongguan economic zone, a leading urban economic development area in China. The findings may provide useful information for policy makers and health service providers in developing programs to optimize health service delivery to rural-to-urban female migrant workers - an underprivileged working population in urban China.

## Methods

### Study design and participants

A cross-sectional survey was carried out in 2013 in three factories in the Shenzhen-Dongguan economic zone in China. The study was aimed to evaluate HRQOL and health service utilization in rural-to-urban women migrant workers in light-duty large factories (>1000 employees) without strong occupational health risk hazards. With an estimated annual hospitalization rate (a primary outcome) of about 6.0%, allowing a maximal deviation of 1.2%, at an alpha error of 5%, a minimal sample size of 1204 subjects was required. The required sample sizes would have been smaller if the sample size calculations were based on other outcomes (2-week physician visit or HRQOL scores). Three factories (a textile manufacturer, a furniture assembler and a camera assembler) were approached and accepted to allow the implementation of the study protocol. Women in the three factories were all light-duty manual workers. The study subjects were selected by a cluster sampling method. We randomly sampled 20 of 61 workshops (about 1/3) in the three factories. All consented eligible subjects in the sampled workshops were recruited (n = 1438 rural–to-urban female migrant workers). Data were collected via face-to-face interview by trained study personnel. The Research Ethics Board of Guangzhou Medical University approved the study. Written informed consent was obtained from the study participant, or from a parent or legal guardian of the participant if her age was <18 years (a few participants of 16–17 years old).

### Procedures

Well-trained interviewers (medical students from Guangzhou Medical University) collected research data through face-to-face interviews using structured study questionnaires. The interviewers received training and engaged in group discussions and simulated interviews to standardize data collection and recording procedures.

### General study questionnaire

The general study questionnaire included information items on socio-demographic characteristics, health-related factors and health service utilization. Socio-demographic variables included age, marital status, education, medical insurance and duration of employment (years). Health-related variables included body mass index (BMI), any diagnosed chronic disease (yes/no), and recent-month self-reported morbidity (diagnosed disease, or self-perceived disease due to symptoms). BMI was calculated as weight/height^2^ (kg/m2), and categorized as “underweight” (<18.5), “normal weight” (18.5-23.9), “overweight” (24.0-27.9), and “obese” (≥28.0), according to the Chinese BMI reference standards [29]. Recent-month self-reported morbidity was obtained in the question “Have you been ill over the past month?”, an indicator of diagnosed disease plus self-perceived disease (due to uncomfortable symptoms). Health service utilization variables included recent two-week physician visit and annual hospitalization. “Recent two-week physician visit” was obtained in the question “In the past two weeks, have you ever visited a doctor?”. Annual hospitalization” was obtained in the question “In the last one year, have you been hospitalized?”.

### SF-36 questionnaire

The Chinese version of the Short Form (SF-36) Health Survey, which was translated from the standard English version of SF-36, showed satisfactory construct and clinical validity and internal consistency for measuring HRQOL [30-32]. The SF-36 was a generic measure of HRQOL in both physical and mental domains [33-37]. The questionnaire comprises 36 questions covering eight domains: four in the area of physical health including physical functioning (PF), role physical (RP), bodily pain (BP) and general health (GH), and four in the area of mental health including vitality (VT), social functioning (SF), role-emotional (RE) and mental health (MH). Both physical and mental health scores have been empirically validated [38,39]. Total score in each of the eight domains can be converted into 0–100 scale; higher scores indicating better HRQOL. SF-36 has been widely accepted as a valid instrument for measuring HRQOL in the general population and in those with specific health conditions due to its sound psychometric properties, brevity and comprehensiveness [40-42].

### Statistical analysis

Statistical analyses were performed using Statistical Package for Social Sciences (SPSS), version 13.0 (SPSS Inc., Chicago, IL). Mean and standard deviation (SD) are presented for continuous variables. Frequency and percentage are presented for categorical variables. Clustered logistic regression [43] was employed to explore the impacts of socio-demographic, health-related and HRQOL factors (three clusters) on health service utilization. The two dependent (outcome) variables were recent two-week physician visit (“no visit”, “at least one visit”) and annual hospitalization (“no hospitalization”, “at least one hospitalization ”). Significance level was set at *P* <0.05 for including predictor variables into the regression models.

We first explored the associations of the eight domains of SF-36 with health service utilization without adjustment for other factors. In subsequent clustered regression analyses, independent predictor variables were grouped into three clusters according to nature of the study variables: Cluster 1, socio-demographic variables; Cluster 2, health-related variables; Cluster 3, HRQOL variables. Health-related variables included BMI, self-reported morbidity and chronic illnesses. HRQOL variables included the standard 8 domains as measured by SF-36. Multidirectional associations may exist between the three clusters of independent variables and the dependent variables. Specifically, cluster 1 may affect cluster 2, cluster 3 and the two dependent variables of health service utilization. Similarly, cluster 2 may impact cluster 3 as well as the two dependent variables, while cluster 3 may affect the dependent variables. As a result, simultaneous consideration of variables from the clusters in a free multiple regression model (i.e. a free forward stepwise logistic regression model) might result in confounded inference. Therefore, clustered logistic regression [43] was adopted to analyze whether the addition of HRQOL variables to the models including socio-demographic and health-related variables could significantly increase the explanatory power of the risk-adjustment models. The two health service utilization variables were regressed on the three clusters of independent variables. The final regression model was determined in three steps: (1) A forward stepwise regression of health service utilization for the cluster 1 variables; (2) A forward stepwise regression for the cluster 2 variables with the equation derived from step 1 as a fixed part of the new regression model; (3) A forward stepwise regression for the cluster 3 variables with the equation derived from step 2 as a fixed part of the new regression model.

The independent effect of each cluster on the dependent variable was calculated by the difference in the corresponding *R*^2^ values between the two regression models (with vs. without the cluster). The independent contribution share of each cluster was calculated as individual *R*^2^ change/total *R*^2^ change in the final model × 100%. In logistic regression models, the *R*^2^ is the Nagelkerke ‘pseudo’ *R*^2^ which is similar to the classical *R*^2^ in linear regression models for data interpretation [43].

Results for the pooled data in the whole study cohort are presented, because similar results were observed among subjects from the three factories in exploratory analyses.

## Results

### Participant characteristics

Descriptive statistics on study variables are presented in Table 1. Subjects aged between 16 and 59 years, with an average of 31.4 ± 9.2 (SD) years. Most participants were 26–35 years of age, married, had worked less than 5 years (88.5%), had completed middle school education, had medical insurance (84.2%), normal weight (64.8%), and didn’t report any chronic disease (91.7%). The recent-month self-reported morbidity was 67.7%. HRQOL scores in the eight domains varied from 70.6 ± 18.5 to 93.8 ± 10.9. General health scored the lowest, while physical functioning scored the highest.

**Table 1:** **Characteristics of study participants (n = 1,438 female rural-to-urban migrant workers) in Shenzhen-Dongguan economic zone, China 2013**

**Variable**	**Mean ± SD**	**N (%)**
**Cluster 1 (social-demographic)**		
Age (years)	31.4 ± 9.2	
16-25		125 (8.7)
26-35		564 (39.2)
36-45		398 (27.7)
≥46		351 (24.4)
Marital status, married (yes)		997 (69.3)
Medical insurance (yes)		1211 (84.2)
Education		
Primary school or lower		290 (20.2)
Middle school		846 (58.8)
High school or above		302 (21.0)
Duration of employment (years)	2.2 ± 3.0	
<5.0		1273 (88.5)
5.0 ~ 9.9		111 (7.7)
≥10.0		54 (3.8)
**Cluster 2 (health-related)**		
Recent-month self-reported morbidity		973 (67.7)
Body mass index (BMI)	22.4 ± 3.4	
<18.5 (Underweight)		139 (9.7)
18.5-23.9 (Normal weight)		932 (64.8)
24.0-27.9 (Overweight)		262 (18.2)
≥28.0 (Obese)		105 (7.3)
Chronic diseases		
0		1318 (91.7)
1 or more		120 (8.3)
**Cluster 3 (HRQOL domain scores)**		
Physical functioning (PF)	93.8 ± 10.9	
Role physical (RP)	85.9 ± 25.6	
Bodily pain (BP)	85.1 ± 16.6	
General health (GH)	70.6 ± 18.5	
Vitality (VT)	71.4 ± 18.0	
Social functioning (SF)	89.9 ± 14.2	
Role-emotional (RE)	82.0 ± 28.1	
Mental health (MH)	78.4 ± 15.2	
**Outcomes**		
Recent 2-week physician visit		
0		1319 (91.7)
1 or more		119 (8.3)
Hospitalization in the last year		88 (6.1)
0		1350 (93.9)
1 or more		88 (6.1)

HRQOL = health related quality of life.

### Associations of HRQOL’s eight domains with health service utilization

Higher HRQOL scores in BP and GH domains were associated with significantly lower odds of recent two-week physician visit (Table 2). That is, the risk of recent two-week physician visit increased significantly with decreasing HRQOL scores in BP and GH domains. The risk of hospitalization during the last 1-year increased significantly with decreasing HRQOL scores in RP and GH domains.

**Table 2:** **Associations of HRQOL domains with health service utilization** ^**a**^

**Predictor**	**Two-week physician**	**Visit**	**Annual**	**Hospitalization**
**Variable**	**OR** ^**b**^ **(95%** **CI)**	***P***	**OR** ^**b**^ **(95%** **CI)**	***P***
PF	1.05(0.87-1.27)	0.632	0.91(0.76-1.10)	0.349
RP	0.97(0.76-1.23)	0.786	0.73(0.56-0.95)	0.019*
BP	0.62(0.50-0.76)	<0.001*	0.93(0.73-1.19)	0.561
GH	0.63(0.50-0.80)	<0.001*	0.62(0.48-0.81)	<0.001*
VT	1.23(0.95-1.60)	0.115	1.21(0.90-1.62)	0.204
SF	0.94(0.77-1.15)	0.560	1.03(0.81-1.31)	0.792
RE	1.07(0.84-1.38)	0.572	1.16(0.87-1.55)	0.304
MH	0.95(0.74-1.22)	0.659	1.02(0.76-1.36)	0.895

HRQOL = health related quality of life; OR = Odds ratio; CI = confidence interval;

PF = Physical functioning; RP = Role physical; BP = Bodily pain; GH = General health;

VT = Vitality; SF = Social functioning; RE = Role-emotional; MH = Mental health.

^a^The eight HRQOL domains as measured by the 36-items Short Form (SF-36) Health Survey were included as predictor variables for recent two-week physician visit or annual hospitalization in a multivariate regression model without adjustment for other variables.

^b^Odds ratio per SD increase in a predictor variable.

*P < 0.05.

### Determinants of health service utilization

In the first cluster (social demographic), all variables were not significantly associated with two-week physician visit (Table 3). In the second cluster (health related variables), recent-month self-reported morbidity and the presence of chronic disease were positively associated with the likelihood of recent two-week physician visit. The independent contribution of health-related variables was 84.4%. The risk of recent two-week physician visit decreased significantly with increasing HRQOL scores in BP (adjusted OR = 0.71 per SD increase, p = 0.001) and GH (adjusted OR = 0.76 per SD increase, p = 0.01) domains. The independent influence of BP and GH on the risk of recent two-week physician visit was 15.6%.

**Table 3:** **Clustered logistic regression models explaining recent 2-week physician visit by socio-demographic characteristics (cluster 1), health-related factors (cluster 2) and HRQOL domains (cluster 3)**

**Predictor variable** ^**b**^	**OR** ^**b**^ **(95**% **CI)**	***P***	**Nagelkerke** ^**c**^ ***R*** ^***2***^	**Independent contribution (%)** ^**d**^
**Cluster 1**				
Total			0	0
**Cluster 2**				
Recent month morbidity	7.56 (3.02,18.92)	<0.001		
Chronic diseases	1.94 (1.27,2.94)	0.002		
Total			0.141	84.4.
**Cluster 3**				
HRQOL- BP^a^	0.71 (0.58,0.87)	0.001		
HRQOL - GH^a^	0.76 (0.61,0.94)	0.010		
Total			0.167	15.6

HRQOL = health related quality of life; OR = Odds ratio; CI = confidence interval;

BP = Bodily pain; GH = General health.

^a^For HRQOL BP and GH scores, odd ratios per SD increase were calculated.

^b^Only variables with *P* < 0.05 were included in the model.

^c^Nagelkerke *R* ^*2*^ is the variance of in the dependent variable (recent 2-week physician visit) explained by all independent variables included in the regression model.

^d^The independent contribution of each cluster of predictors to the variation in two-week physician visit was calculated as individual corresponding *R* ^2^ change/total *R* ^2^ change in the final model × 100%.

In the first cluster, social demographic variables, marital status and duration of employment were associated with annual hospitalization (Table 4). The independent contribution from socio-demographic variables was 78.8%. In the second cluster, surprisingly, health-related variables were not associated with annual hospitalization. The risk of annual hospitalization decreased significantly with increasing HRQOL scores in RP (adjusted OR = 0.75 per SD increase, p = 0.003) and GH (adjusted OR = 0.64 per SD increase, p < 0.001) domains. The independent influence of RP and GH on the risk of annual hospitalization was 21.2%.

**Table 4:** **Clustered logistic regression models explaining annual hospitalization by socio-demographic characteristics (cluster 1), health-related factors (cluster 2) and HRQOL domains (cluster 3)**

**Predictor variable** ^**b**^	**OR** ^**a**^ **(95% CI)**	***P***	**Nagelkerke** ^**c**^ ***R*** ^***2***^	**Independent contribution (%)** ^**d**^
**Cluster 1**				
Marital status	5.27 (2.48,11.18)	<0.001		
Employment duration (y)	1.08 (1.02,1.14)	0.009		
Total			0.093	78.8
**Cluster 2**				
Total			0.093	0
**Cluster 3**				
RP	0.75 (0.62,0.91)	0.003		
GH	0.64 (0.52,0.80)	<0.001		
Total			0.118	21.2

HRQOL = health related quality of life; OR = Odds ratio; CI = confidence interval;

RP = Role physical, GH = General health.

^a^For RP and GH scores, odd ratios per SD increase were presented.

^b^Only variables with *P* <0.05 were included in the model.

^c^Nagelkerke *R* ^*2*^ is the variance in the dependent variable (annual hospitalization) explained by all independent variables included in the regression model.

^d^The independent contribution of each cluster of predictors to the variation in annual hospitalization was calculated as individual corresponding *R* ^2^ change/total *R* ^2^ change in the final model × 100%.

## Discussion

### Main findings

To the best of our knowledge, this is the first study demonstrating an association between HRQOL and health service utilization in Chinese rural-to-urban female migrant workers. The results revealed that health service utilization increased modestly and significantly with decreasing scores in certain HRQOL domains. Nevertheless, the independent influence of the HRQOL was smaller than that of socio-demographic variables on annual hospitalization, and than that of health-related variables on recent 2-weeks physician visit. The strong association of hospitalization with socio-demographic variables and the lack of association with health related variables suggest that affordability may be a major determinant in the use of hospitalization care for female migrant workers.

### Comparisons with previous studies

We found that HRQOL was a significant independent predictor of health service utilization in Chinese female rural-to-urban migrant workers. This finding is consistent with the findings in previous reports on the impact of HRQOL on health service utilization in other study populations [13,23-25,44]. In the USA, Nelson and colleagues reported that physical functioning and mental health were two important predictors of both clinic consultation and hospitalization in patients with chronic diseases [23]. Dominick and colleagues reported that HRQOL, especially pain frequency, could be an invaluable tool for evaluation of future health service utilization among patients with osteoarthritis [25]. In northern Europe, Miilunpalo and colleagues found that HRQOL was associated with annual outpatient consultation in a working-age population in Finland [44]. In Hong Kong, Lam CL and colleagues reported that HRQOL was a more important determinant of outpatient consultation than chronic morbidity and socio-demographic factors [24]. In contrast, we found that the independent influence of HRQOL on health service utilization was smaller than that of social-demographic or health related variables. The differential findings may be due to different study context –Hong Kong is quite different from mainland China in socioeconomic environment and health care system. In China, Chen T and colleagues showed that the number of monthly outpatient consultations increased significantly with decreasing HRQOL scores in PF, BP and GH domains, while annual hospitalization rate increased significantly with decreasing scores in PF and GH domains in primary care patients [13]. In contrast, among female migrant workers, our study showed that the likelihood of two-week physician visit increased significantly with decreasing scores in BP and GH domains, while the risk of annual hospitalization increased significantly with decreasing scores in RP and GH domains. Therefore, our findings are consistent with those of Chen’s study with respect to the impact of GH on health service utilization, but somewhat different with respect to the roles of PF and RP domains. The differential findings might be due to different study populations: primary care patients in Chen T’s study vs. mostly healthy rural-to-urban migrant workers in our study. Besides, all the 4 domains - physical functioning (PF), role physical (RP), bodily pain (BP) and general health (GH) are the measures of physical health [38]. Taken together, it seems that physical health domains as measured by SF-36 are important predictors of health service utilization in Chinese population. In both studies, the independent influence of the SF-36 domains on health service utilization was observed to be smaller than that of other clusters of variables (social-demographic or health- related). Similar to the findings in Zhu CY’s study [27], the observed HRQOL scores in most domains (PF, BP, GH, VT, SF) were lower in rural-to-urban migrant female workers in our study population as compared to the norms for Chinese women [32].

### Limitations

Self-reported information is prone to inaccuracies. Nelson and colleagues reported that 5% of subjects over-reported clinic consultations over the last one month, and that self-reported health service utilization could not be taken as actual health service utilization, but only as an indicator of utilization pattern [23]. We expected that such errors in self-reports were random and would not affect the validity of the comparisons. We have data on education, but no data on income which is another important dimension of socioeconomic status associated with HRQOL [45]. Income reporting is a sensitive issue and highly unreliable in China. Nevertheless, it is known that rural-to-urban migrant light-duty manual workers are low-pay workers in urban areas. In addition, we have data on duration of employment which may partly reflect their relative income levels. Our findings are suggestive rather than definitive. We could not draw a firm causal inference from an observational study. HRQOL, health status and health service utilization are inter-correlated, and it is difficult to disentangle causes vs. effects. We do not have detailed information on the specific diseases the study subjects had at the time of investigation, reasons of hospitalization, types of insurance, cost for health service utilization and burdens from out-of-pocket payment. It would be of interest to have these variables in future studies for a more in-depth understanding on the association between HRQOL and health service utilization in Chinese female rural-to-urban migrant workers. The study was based on a sample of rural-to-urban migrant women workers in an affluent economic region. We could not assume that the findings are applicable to other regions without additional validation studies. Nevertheless, it is likely that similar associations may be observed in female rural-to-urban migrant workers in other urban areas in China as they share some similarities in much lower socioeconomic status as compared to local urban residents.

## Conclusions

Lower HRQOL scores in bodily pain, role physical and general health domains were associated with more frequent health service utilization in Chinese female rural-to-urban migrant workers. The findings suggest that HRQOL may have a modest influence on health service utilization in this underprivileged population in urban China.

## Abbreviations

HRQOL: Health-related quality of life
PF: Physical functioning
RP: Role-physical
BP: Bodily pain
GH: General health
VT: Vitality
SF: Social functioning
RE: Role-emotional
MH: Mental health
OR: Odds ratio
CI: Confidence interval
